# Therapy resistance mediated by exosomes

**DOI:** 10.1186/s12943-019-0970-x

**Published:** 2019-03-30

**Authors:** Teresa Bernadette Steinbichler, József Dudás, Sergej Skvortsov, Ute Ganswindt, Herbert Riechelmann, Ira-Ida Skvortsova

**Affiliations:** 1 Department of Otorhinolaryngology, Head and Neck Surgery, Medical University of Innsbruck, Innsbruck, Austria; 2 Laboratory for Experimental and Translational Research on Radiation Oncology (EXTRO-Lab), Department of Therapeutic Radiology and Oncology, Medical University of Innsbruck, Anichstr. 35, A-6020 Innsbruck, Austria; 3 EXTRO-Lab, Tyrolean Cancer Research Institute, Innsbruck, Austria

**Keywords:** MDR genes, Apoptosis, Cancer stem cells, Immune surveillance, Dormancy, EMT

## Abstract

Therapy resistance can arise within tumor cells because of genetic or phenotypic changes (intrinsic resistance), or it can be the result of an interaction with the tumor microenvironment (extrinsic resistance). Exosomes are membranous vesicles 40 to 100 nm in diameter constitutively released by almost all cell types, and mediate cell-to-cell communication by transferring mRNAs, miRNAs, DNAs and proteins causing extrinsic therapy resistance. They transfer therapy resistance by anti-apoptotic signalling, increased DNA-repair or delivering ABC transporters to drug sensitive cells. As functional mediators of tumor-stroma interaction and of epithelial to mesenchymal transition, exosomes also promote environment-mediated therapy resistance.

Exosomes may be used in anticancer therapy exploiting their delivery function. They may effectively transfer anticancer drugs or RNAs in the context of gene therapy reducing immune stimulatory effects of these drugs and hydrophilic qualities facilitating crossing of cell membranes.

## Introduction

Tumor therapy resistance is defined as reduction of effectiveness of an antineoplastic therapy. Therapy resistance is one of the major obstacles in cancer treatment. Therapy resistance can arise within tumor cells because of genetic or phenotypic changes (intrinsic resistance), or it can be the result of the tumor microenvironment protecting tumor cells against treatment (extrinsic resistance). Thus, extrinsic resistance arises from the interaction between tumour cells and their surrounding [1–3]. This extrinsic resistance can even cause changes in gene expression profiles by the exchange of small RNAs, like microRNAs (miRNAs) [4].

**Fig. 1:**
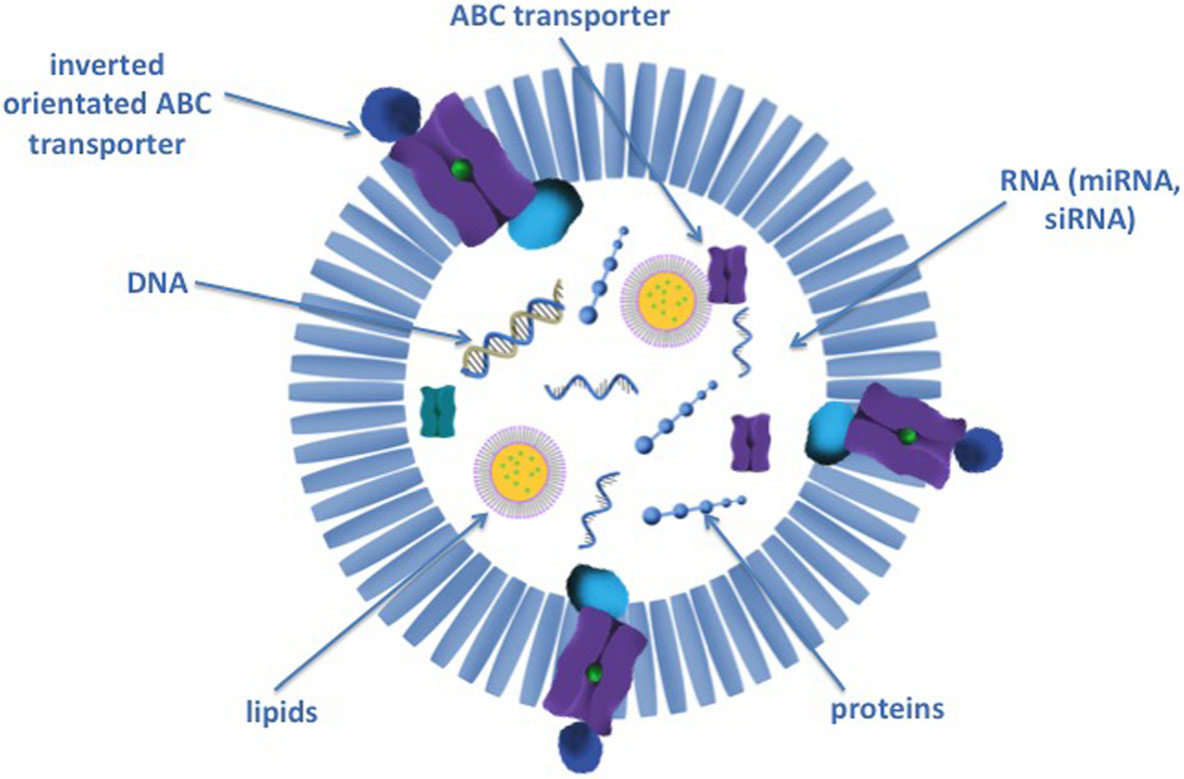
Exosomal cargo that mediates therapy resistance. Exosomes cause therapy resistance in the recipient cell by transporting DNA, RNA (micro RNA, short interfering RNA), lipids and proteins. They cause decreased apoptosis and anti tumor immunosurveillance and increased DNA repair and stemness in recipient cells. Furthermore they transport multidrug resistance (MDR) transporter to recipient cells or integrate them in reverse orientation in their membrane to decrease intra- and intercellular drug concentration

Exosomes are nanosized membrane vesicles that constitutively released by almost all cell types. The main physiological role of the exosomes is to mediate cell-cell communication by transferring messenger RNAs (mRNAs), miRNAs, DNAs and proteins (Fig. 1) [1].

Exosomes can transport RNAs from one cell to another causing alterations in protein expression of the recipient cell (Fig. 1). Normally RNAs are rapidly cleaved in the blood stream by RNAses [4]. Exosomes protect RNAs from cleavage and allow exchange of RNAs by different cells even over long ranges or different organs [5]. Exosomes reduce hydrophilic qualities of RNAs and therefore facilitate crossing of cell membranes [6].

Furthermore exosomes can mediate therapy resistance by distributing proteins that increase tumor cell survival and DNA repair (Fig. 1) [3]. Exosome signalling generates favourably therapy resistant conditions in the tumor microenvironment and induces the generation of cancer stem cells (CSC) through epithelial-mesenchymal transition (EMT) [7, 8].

**Fig. 2:**
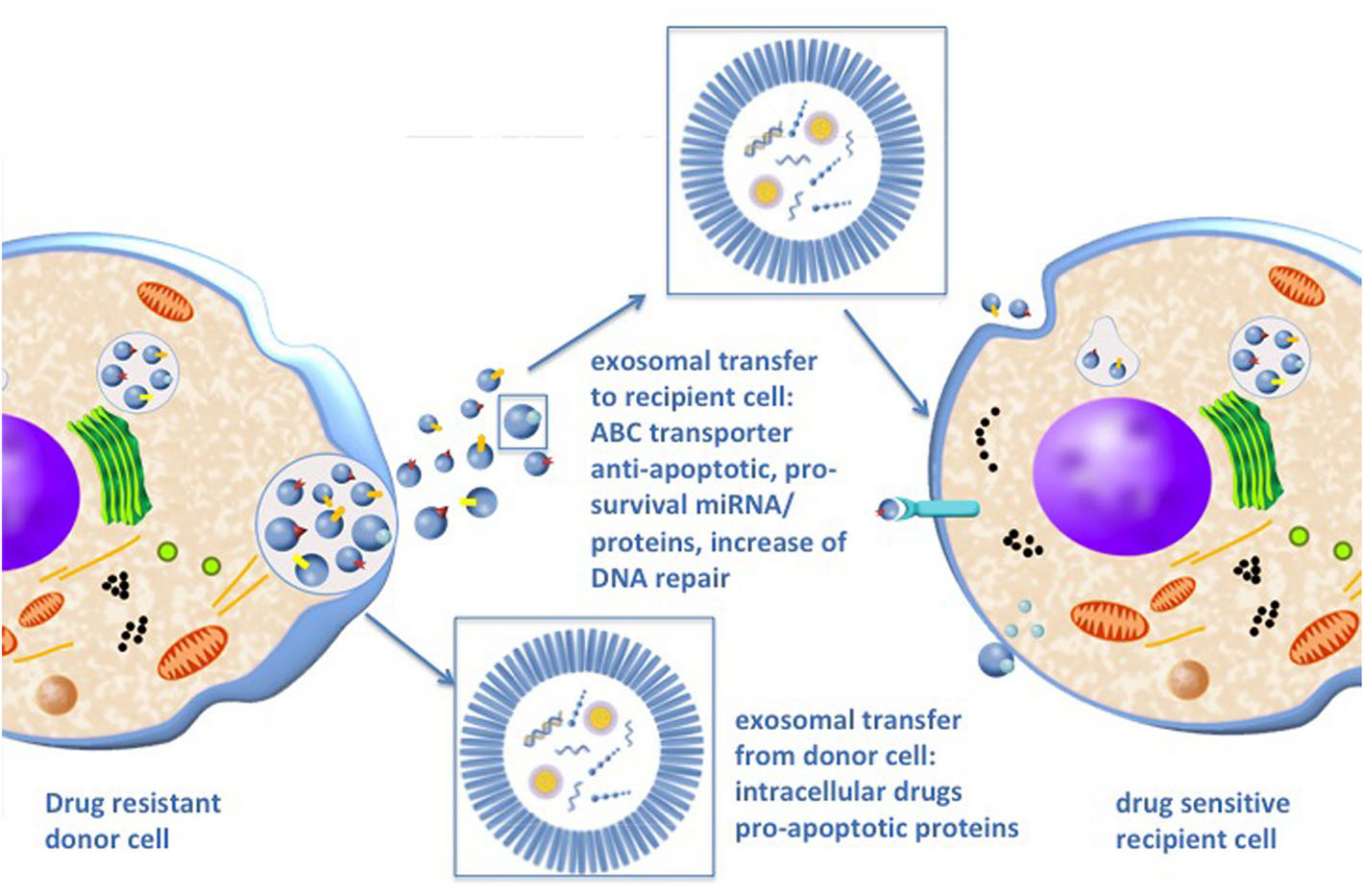
Exosomes cause therapy resistance in recipient and donor cells Exosomes transport multi drug resistance (MDR) transporter to recipient cells causing increased efflux of drugs by integrating MDR transporter in the cell membrane. Furthermore, exosomes cause increased anti-apoptotic signaling and DNA repair in the recipient cell. Besides these effects on recipient cells exosomes are important for therapy resistance of the donor cell as well. MDR transporters are incorporated into the exosomal membrane with reverse orientation, which promotes the influx of drugs from the donor cell into the exosome reducing intracellular drug concentration. Exosomes also reduce intracellular concentration of pro-apoptotic signalling factors by transporting them away from the donor cell

Of note, besides the effects of exosomes on recipient cells, exosomes increase therapy resistance of the donor cell by reducing intracellular drug concentrations and by disposing pro-apoptotic proteins like caspases (Fig. 2) [9].

The following review summarizes all the mentioned aspects of exosome-mediated therapy resistance of tumor cells. A systematic literature search was performed using PubMed and Cochrane with the combination of the following keywords: “therapy resistance”, “exosomes” and “tumor”.

## Tumor therapy resistance through decreased inter- und intracellular drug concentrations

### Background

Tumor cells with acquired chemotherapy resistance often display features of multiple drug resistance (MDR). MDR is an insensitivity of cancer cells not only to previously used drugs but also to many other drugs with different chemical structure and mechanism of action [10].

### Direct transfer of drug transporters

MDR is associated with increased expression of drug transporters from the adenosine triphosphate (ATP)-binding cassette transporter (ABC) family. These proteins use energy from ATP hydrolysis for active removal of drugs from cells preventing accumulation of anti-cancer drugs [11]. The multidrug resistance protein 1 gene (*MDR1*, *ABCB1*) encodes for the most important drug transporter, p-glycoprotein (P-gp). Expression of this protein was noted in over 50% of cancers with MDR phenotype where it can be constitutively expressed or induced by chemotherapy [12]. Approximately 20 different cytotoxic drugs including paclitaxel and doxorubicin are substrates to this transporter. The second most important drug transporter is breast cancer resistant protein (BCRP) encoded by the *ABCG2* gene [13].

There is substantial experimental evidence that P-gp and other MDR transporters can be transferred from drug resistant to drug sensitive tumor cells by circulating exosomes [14] causing acquired therapy resistance of the recipient cells in vivo and in vitro (Fig. 2) [14–16]. Mechanistically, functional P-gp is incorporated in the exosomal membrane and transferred to donor cells who in return integrate it in their cell surface [14]. Corcoran and colleagues demonstrated in an in vitro model of prostate cancer that MDR1/P-gp is transported via exosomes to docetaxel sensitive cells leading to acquired docetaxel resistance [17]. Drug-sensitive breast cancer cells were shown to acquire a drug-resistant phenotype after exposure to exosomes extracted from a drug resistant cell line. Furthermore, the observed increase in P-gp levels of the recipient cells was proportional to the amount of releases exosomes from drug-resistant cells [18]. In vivo studies of a neuroblastoma xenograft mouse model confirmed this exosomal P-gp transfer and even indicated a higher efficiency of this exosomal transfer under physiological conditions than in cell cultures [15].

### Modulation of MDR gene expression by exosomal miRNA/mRNA transfer

Levchenko and colleagues demonstrated that exosomal P-gp transfer led to a prolonged acquired resistant phenotype of tumor cells characterized by the P-gp expression for up to 4 months [15]. The transfer of P-gp alone cannot explain these observed long-term effects, since the half-life of P-gp is approximately 14–17 h [16]. Recent experiments suggested that P-gp-related miRNAs and even mRNAs transferred by exosomes can cause a long-term P-gp expression in the recipient cells [16]. MiR-451 and miR-27a, which are both enriched in exosomes from drug resistant cells [16], upregulate P-gp expression explaining these long-term effects [16, 19]. Furthermore, transcription of exosomal delivered mRNAs contribute to the activation of nuclear factor kappa B (NF-κB), which is known to be involved in the induction of drug resistance by increased MDR1 expression [20].

### Reduction of intra- and intercellular drug concentration by exosomes

In addition to their role in conferring therapy resistance to recipient cells, exosomal ABC transporters contribute to drug-resistance of the donor cell by sequestering drugs in exosomes, thereby reducing intracellular drug concentration (Fig. 2). Therefore, P-gp is incorporated into the exosomal membrane with reverse orientation, which promotes the influx of drugs from the donor cell into the exosome [16, 21]. ABCG2-rich exosomes are able to take up riboflavin, topotecan, imidazoacridinone and methotrexate in the same way [22]. Exosomal ABCG2 expression can be induced by the phosphoinositide-3-kinase–protein kinase B (PI3K)- protein kinase B (Akt) signaling pathway and inhibition of this pathway led to cytoplasmic re-localization of ABCG2 and increased drug sensitivity in breast cancer cells [23]. This sequestration of cytotoxic agents appears to be pH dependent as the cisplatin transport into exosomes is increased in an acidic microenvironment [24]. Acidification is common in tumors due to the so-called “Warburg effect” with high extracellular lactate content and inadequate neovascularization [24–26]. Additionally, many tumors express H + -ATPases, which pump protons across the plasma membrane and contribute to the acidification of the tumor microenvironment. Basic chemotherapeutic drugs are trapped in the acidic exosomes [25].

Exosomes can also reduce extracellular drug levels by displaying bait targets for therapeutic antibodies on their surface (Fig. 2). Exosomes carry e.g. the cluster of differentiation (CD)-20 receptor, which acts as a bait for therapeutic anti-CD20 antibodies such as rituximab [27]. In breast cancer cells, the human epidermal growth factor receptor-2 (HER2) is found on the surface of exosomes, resulting in the sequestering of the therapeutic monoclonal antibody Herceptin®. Thus, exosomes protect breast cancer cells from antibody-dependent cell-mediated cytotoxicity (ADCC) by NK cells [28]. Advanced breast cancer is associated with increased exosome secretion and increased exosome binding to Herceptin®, suggesting that exosomes facilitate cancer progression by limiting drug availability [28]. Similar results were observed in epithelial cell adhesion molecule (EpCam)-positive breast cancer cells with the EpCam-specific antibody C215 [29].

## Tumor therapy resistance through exosome-mediated interference with cell cycle and DNA repair

### Background

Exosome-mediated reduction of intracellular and extracellular concentrations of chemotherapeutic agents cannot explain exosome-mediated irradiation resistance. Exosomes can induce irradiation resistance and chemotherapy resistance by influencing cell cycle regulation, apoptosis, and DNA repair of tumor cells [5, 30].

### Change in apoptotic homeostasis

Exosomes can shift cellular homeostasis between anti- and pro-apoptotic signals, resulting in increased survival of tumor cells following exposure to DNA-damaging drugs or irradiation. Exosomes can promote tumor cells survival by either decreasing pro-apoptotic signalling in the donor cell or by increasing anti-apoptotic signalling in the recipient cells (Fig. 2) [9, 31].

To promote survival of donor cells, exosomes can reduce intracellular levels of pro-apoptotic proteins shifting the intracellular balance to an anti-apoptotic state [32]. The release of caspase-3 containing exosomes prevented the induction of apoptosis in donor cells. Conversely, the inhibition of this release resulted in an intracellular accumulation of caspase-3 and consequently apoptosis in endothelial donor cells [32].

In recipient cells, exosomes can promote tumor cell survival by three important anti-apoptotic mechanisms:

1. Exosomes could stimulate recipient cells through surface expressed receptors to induce signal transduction and the activation of anti-apoptotic pathways. In vivo and in vitro studies of multiple myeloma demonstrated that bone-marrow derived exosomes contain high levels of interleukine-6 (IL-6) [33] and this interaction of exosomal IL-6 with multiple myeloma cells inhibited tumor cell apoptosis [2, 33, 34].
2. Exosomes may transfer receptors like CD41 to target cells. CD41 (integrin α-IIb) binds to extracellular matrix, causing integrin-mediated inhibition of apoptosis by preventing anoikis [9, 31, 35].
3. Exosomes could directly transfer transcriptional factors and induce the activation of anti-apoptotic or pro-survival pathways. In a murine model of multiple myeloma, bone marrow derived exosomes inhibit cleavage of full-length caspase 3 and 9 and consequently apoptosis. Furthermore they promoted tumor cell survival via inhibition of the c-Jun N-terminal kinase (JNK) pathway. This led to Bortezomib resistance [31]. Other involved anti-apoptotic signalling pathways in exosome mediated therapy resistance are p38, p53, JNK, Rapidly Accelerated Fibrosarcoma (Raf)/ Mitogen-activated protein kinase kinase (MEK)/ extracellular signal–regulated kinases (ERK) and Akt [9, 31, 36].

### Anti-apoptotic signalling mediated by miRNA

Exosomes can confer resistance to therapy-sensitive tumor cells by transmitting miRNAs that alter cell cycle control and induce anti-apoptotic programs (Fig. 1). MiRNAs are small, non-coding RNAs with a length of 18 to 24 nucleotides that control gene expression posttranscriptionally [37]. They accumulate in exosomes where they are protected from cleavage by RNAses in the blood [4]. Tumor exosomes do not only transport RNAs but can even modify miRNAs to e.g. induce tumor progression in breast cancer and perform cell-independent miRNA synthesis [38]. Chen and colleagues demonstrated miR-222 was upregulated in exosomes from drug resistant breast cancer cells. MiR-222 conferred this resistance to drug sensitive cells by down regulation of the Phosphatase and tensin homolog (PTEN) pathway, which promotes cell cycle arrest [39].

Furthermore, exosomes can increase therapy resistance of the donor cell by decreasing intracellular levels of tumor suppressive miRNAs [40]. The release of exosomes containing tumor-suppressive miR-145/−34a from colorectal cancer cells led to increased 5-fluoruracil resistance of these cells by decreased apoptosis [40].

MiRNA research is challenging because every miRNA influences different pathways by transcriptional regulation and there are thousand different variants. To help understand circulatory miRNAs and their function, databases such as miRandola were developed [41]. The miRandola database contains 3282 entries to date in total and 1106 entries about exosomal mi-RNA. Besides miRNA, this database classifies extracellular circulating RNAs like long non-coding RNA (lncRNA) and circular RNA (circRNA). The miRandola is available online at: http://mirandola.iit.cnr.it/index.php.

A selection of important miRNAs involved in exosomal mediated therapy resistance is summarized in Table 1.

**Table 1:** Exosomal miRNAs involved in therapy response

miRNA	Effects	Cancer type	Reference
Pro-oncogenic			
miR-222	PTEN inhibition- > anti-apoptotic p27/kip inhibition- > cell cycle progression	breast cancer gastric cancer non-small lung cancer hepatocellular carcinoma	[104–108]
miR-485-3p	increased transcription of topoisomerase IIα, multidrug resistance gene 1, cyclin B2- > increased survival, drug resistance, DNA repair	prostate cancer rhabdomyosarcoma lymphoblastic leukemia	[109, 110]
miR-100	interference with HIF1α signalling anti-apoptotic	breast cancer melanoma	[39, 111]
miR-23a	activation of MAPK signalling/inhibition of Sprouty2- > therapy resistance, migration, invasion	breast cancer	[39, 112]
Anti-oncogenic			
miR-145	growth inhibition- > down-regulation of c-myc and Erk5	colorectal cancer B-cell lymphomas gastric cancer	[40, 113–116]
miR-34a	pro-apoptotic inhibition of cKit/ stemness markers (CD44, BMI-1)	colorectal cancer prostate cancer	[117–119]
let 7 a-c	regulates RAS oncogene	lung cancer breast cancer	[108, 120–123]
miR-15/16	inhibition of bcl2/ CDK1/CDK2 expression	leukemia (CLL) prostate cancer	[123–125]

### DNA repair

Exosomes can promote tumor cell survival after genotoxic stress like irradiation by triggering DNA repair. After exposure to irradiation, breast cancer exosomes led to an increased phosphorylation of ataxia telangiectasia mutated (ATM), Histone H2AX and checkpoint kinase 1 (Chk1) in recipient cells indicating the induction of DNA damage repair responses [42]. In an in vitro model of head and neck cancer, tumor derived exosomes were able to increase radioresistance in neighbouring cells by induction of DNA double strand break repair. Furthermore, irradiated tumor cells released more exosomes than un-irradiated cells [43, 44]. Destabilization of the exosomes decreased radioresistance and DNA double strand break repair in recipient cells [44]. As the treatment with RNAse abrogated the exosomal effect on radioresistance, the authors concluded that RNA, especially miRNA, might mediate the observed effect of exosomes on radioresistance [44]. This highlights again the outstanding importance of exosomes for RNA signalling. Interestingly, irradiated cells also take up exosomes more effectively by co-localizing CD29 and CD81 [43].

Exosomes can further increase radiation resistance by promoting cell migration causing cancer cells to leave the irradiated area. In glioblastoma cells, Arscott and colleagues observed that radiation affects the molecular composition of exosomes to adopt a migratory phenotype [45].

## Exosomes and tumor immune escape mechanism

**Fig. 3:**
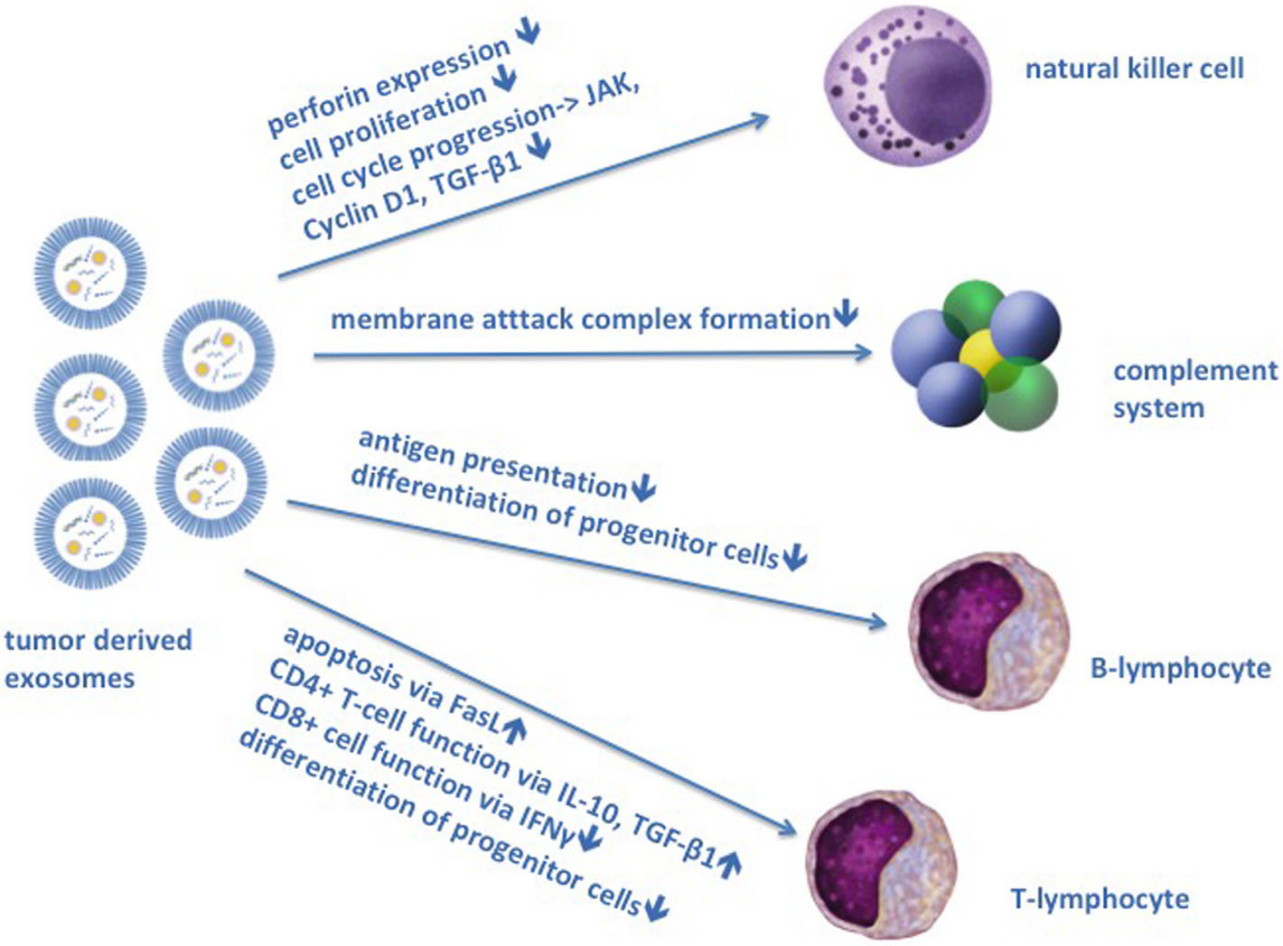
Exosomes and tumor immunosurveillance. Exosome reduce tumor immunosurveillance by interfering with the innate and adaptive immune system. This can cause failure of tumor immunotherapy. Exosomes reduce antibody dependent cytotoxicity by inhibiting natural killer cells. Exosomes reduce activation of the complement systems causing reduced membrane attack complex (MAC) formation and cell lysis. Both mechanism are important effector functions of therapeutic antibodies. Furthermore exosomes reduce T- and B-lymphocyte function and their differentiation from progenitor cells. Lymphocyte function is important for the vaccine effect of therapeutic antibodies

Tumor cells carry molecules on their surface that can be detected by the immune system, known as tumor antigens. Tumor antigens stimulate the immune system of the patient toward an anti-tumor immune response. This fact is exploited by cancer immunotherapy, which aims at increasing patients’ anti-tumor immune response. Especially immune checkpoint inhibitors, like programmed death-ligand 1 (PD-L1) or chemokine receptor type 4 (CXCR4)-inhibitors, and targeted antibodies are under current scientific focus [46]. For successful tumor progression, tumors need to develop immune resistance mechanisms. Exosomes can inhibit tumor immune response and limit the effectiveness of immunotherapy (Fig. 3) [47, 48].

### Innate immune system (NK cells, complement)

Natural killer (NK) cells are key players in antibody dependent cell-mediated cytotoxicity (ADCC) which is suggested as key effector function of therapeutic antibodies [49]. Tumor derived exosomes inhibit NK cell function by decreasing perforin expression and NK cell proliferation (Fig. 3). Exosomes inhibit NK cell cycle progression through Janus kinase 3 (Jak3) and cyclin D1 blockade [50] or via transforming growth factor- β1 (TGF-β1) signalling [51]. Another mechanism of monoclonal antibody therapies is the activation of the complement systems causing membrane attack complex (MAC) formation and cell lysis [52]. Tumor-derived exosomes were demonstrated to contain protein kinase casein kinase 2 (CK2) which phosphorylated complement C9 and protected B-lymphoma cells from complement-mediated lysis [53]. Furthermore, tumor cells can protect themselves from complement-mediated lysis by shedding MACs from their plasma membrane via exosomes [54, 55].

### Adaptive immune system

Lymphocyte effector function can be impaired by treatment with tumor exosomes from different cancer cells. These tumor exosomes reduced production of Interferon-γ and impaired cytotoxic CD8+ T-lymphocyte function [51]. Tumor derived exosomes express the T-cell apoptosis-inducing molecule Fas Ligand (FasL) in vivo and in vitro causing apoptosis of cytotoxic CD8+ T-lymphocytes [56–60]. Besides that, exosomes impair the adaptive immune system by promotion of regulatory T-cell proliferation via TGF-β1 and interleukin-10 (IL-10) at the expense of other T cell subsets [61, 62].

Tumor derived exosomes inhibit differentiation of bone marrow derived progenitor cells to dendritic cells impairing tumor antigen presentation and consequently further T- and B-cell activation [63].

So far, it has been thought that therapeutic antibodies destroy tumor cells by innate immune-effector mechanisms. More recently, it has been observed that therapeutic antibodies can induce long lasting tumor adaptive immunity, which could be responsible for sustained clinical responses. This effect has been termed the vaccine effect of antibodies. It is believed that this effect is caused by the induction of an adaptive immune memory response via T- and B-lymphocytes (Fig. 3) [64]. Exosomes may interfere with this therapeutic adaptive immune response by affecting T- and B- lymphocyte function as well as antigen presentation [65].

## Exosomes and cancer stem cells

### Cancer stem cells and dormancy

Chemotherapy and irradiation are mainly effective against proliferating cells. Tumor cells may enter a quiescent state and thus evade antineoplastic treatment [66]. Cellular dormancy means that cells are recruited in to the G0-phase of the cell cycle but remain capable of cell division in response to mitotic stimulation. Dormancy might also critically contribute to early stages of tumor development and the formation of clinically undetectable metastatic foci [66]. Stroma-derived exosomes induced dormancy in breast cancer cells in vitro and in vivo and this dormancy was associated with an increased carboplatin resistance. This effect was mediated by miR-222/223 and blocking of these miRNAs abrogated dormancy and the associated carboplatin resistance [67].

**Fig. 4:**
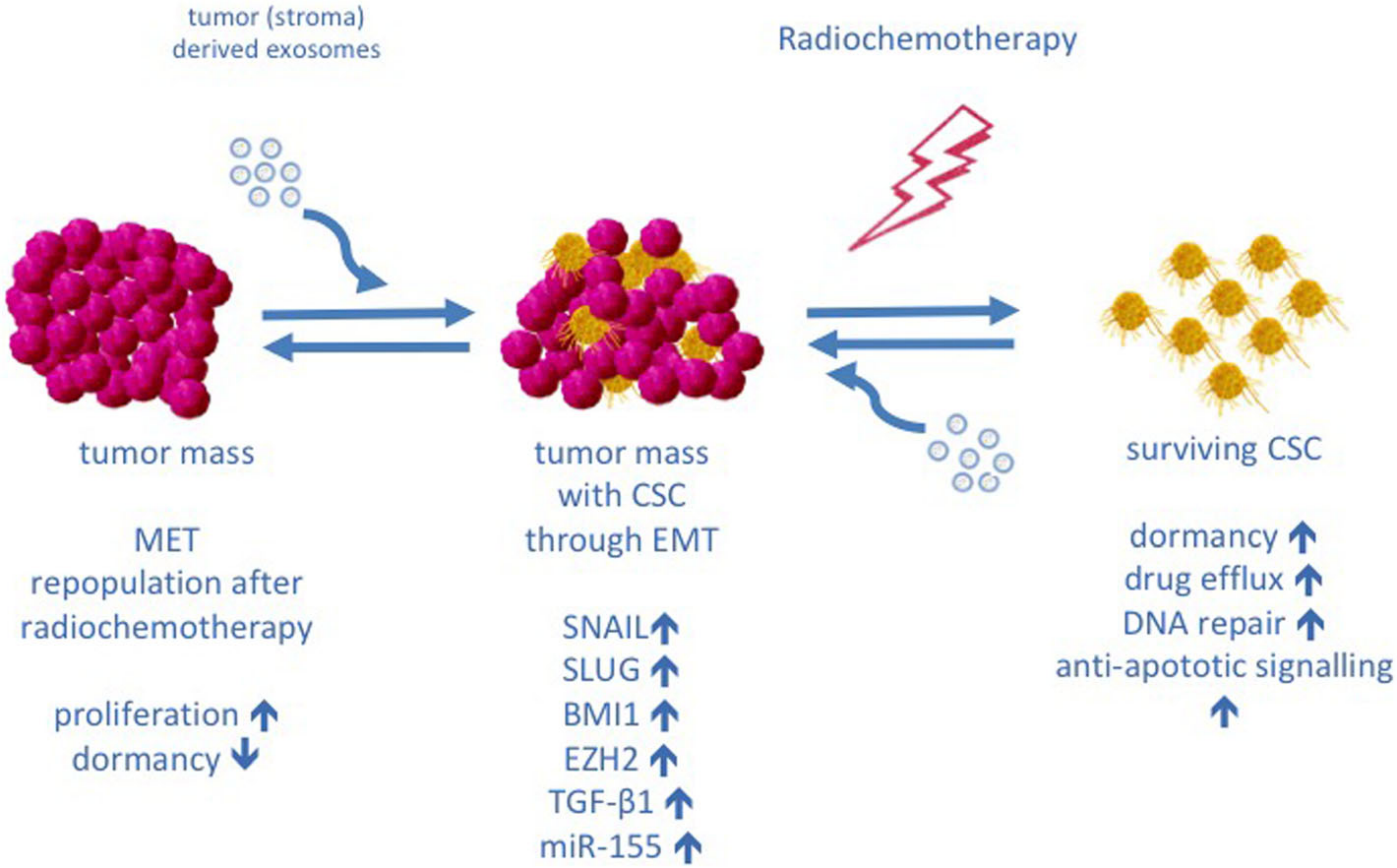
Exosomes, cancer stem cells and EMT. Exosomes induce a cancer stem cell (CSC)-like phenotype in tumor cells through epithelial to mesenchymal transition (EMT). CSC are considered therapy resistant by increased drug efflux capacities and increased DNA repair. A key feature of CSC is their ability to become dormant and thus evade therapy, which is manly effective against proliferating cells. Cellular dormancy means that cells are recruited in to the G0-phase of the cell cycle but remain capable of cell division in response to mitotic stimulation. After therapy CSC can cause repopulation of the tumor mass under the influence of exosomes by undergoing mesenchymal to epithelial transition (MET)

Cellular dormancy is a typical feature of stem cells to maintain tissue homeostasis. In this context, cancer stem cells (CSC) are of particular interest [68]. There is a multitude of literature that stem cells, especially mesenchymal stem cells, can induce drug resistance in tumor cells [69, 70]. Exosomes can induce a CSC like phenotype in tumor cells (Fig. 4) [71]. In an in vitro model of diffuse large cell B cell lymphoma exosomes induced a CSC like phenotype and dormancy through Wingless-related integration site (Wnt)-signalling. These cells expelled doxorubicin more effectively than non-CSC [72]. Boelens and colleagues demonstrated in an in vitro and in vivo model of breast cancer that fibroblast derived exosomes induce a CSC like phenotype in breast cancer cells by Neurogenic locus notch homolog protein 3 (Notch3)/ Signal transducer and activator of transcription 1 (STAT1) signalling which is associated with radiochemotherapy resistance [73]. Exosomal IL-6, Activin-A and granulocyte colony stimulating factor (G-CSF) induced de-differentiation of lung carcinoma cells to a more CSC-like phenotype and reduced cell cycle progression, which was associated with higher methotrexate resistance [74]. Besides promoting CSC-like phenotypes and dormancy in cancer cells, exosomes from fibroblasts can reverse this dormant phenotype by transferring mitochondrial DNA and inducing oxidative phosphorylation allowing recurrence of the disease and metastasis [75].

### Epithelial–mesenchymal transition (EMT)

The acquisition of a CSC like phenotype is closely linked to EMT (Fig. 4) [76]. CSCs may originate from epithelial cells undergoing EMT, a process characterized by loss of E-cadherin expression. EMT is enabled by transcriptional repressors such as SNAIL and SLUG. These events are accompanied by an increase of stemness-related transcription factors, B lymphoma Moloney murine leukemia virus insertion region 1 homolog (BMI1) and enhancer of zeste homolog 2 (EZH2), which may trigger the transformation of epithelial cells into mesenchymal state with the ability to invade other tissues and increased therapy resistance [77–79]. Exosomes are regarded important mediators of these phenotypic changes and tumor stroma interaction [71, 80]. EMT mediates therapy resistance by induction of a dormant, CSC like phenotype [76, 81] and by interaction with anti-apoptotic pathways and DNA repair [79, 82]. Tumor-derived exosomes can force other cancer cells to acquire a mesenchymal phenotype [83]. Exosome-depleted cancer cells failed to gain a stroma-mediated growth advantage, and EMT, mediated by exosomal TGF-β1, was significantly impaired in these cells [84]. Exosomal miR-155 is linked to the development of drug resistance in breast cancer [85, 86]. MiR-155 is also closely involved with TGF-β-induced EMT, invasion, and metastasis demonstrating the link between EMT, CSC, exosomes and therapy resistance [87, 88]. This is supported by the finding of increased CSC markers in tumor biopsies from patients with breast cancer after treatment with chemotherapy for 12 weeks [89].

## Exosomes as therapeutic tumor approach

Despite all the challenges associated with the exosome use for successful cancer treatment, they can also be exploited for the development of new therapeutic techniques.Exosomes may serve for delivery of anticancer drugs or the transfer of RNAs in the context of gene therapy [80]. As exosomes naturally carry RNA between cells, these particles might be useful in gene cancer therapy to deliver therapeutic RNAs, like short interfering RNA (siRNA) or miRNA to target cells. Normally, exogenous RNA is prone to degradation via RNAse, has a limited ability to cross cell membranes due to the negative charged surface and may induce an immune response. Exosomes can overcome these limitations of RNA based therapies [6]. Other advantages of exosomes are their biocompatibility, non-cytotoxicity, low immunogenity and that they are simple to produce, easy to store, have a long life span, and high cargo loading capacity [90–92]. Their small size enables exosomes to easily escape from lung clearance and pass through the blood-brain barrier [93, 94]. Exosomes further enable specific targeting of tumor cells or CSC via surface receptors reducing negative side effects on healthy tissue [92]. These characteristics together make exosomes a promising drug carrier for cancer treatment [92].

Exosomes from mesenchymal stem cells (MSC) can be transfected with synthetic miRNAs. These exosomes reduce chemotherapy resistance [95]. Lou and colleagues transfected adipose tissue-derived MSCs with miR-122. This miR-122 was secreted with exosomes and increased chemotherapy sensitivity of hepatocellular carcinoma cells [96]. Furthermore, MSC can transfer anti-miR-9 via exosomes to glioblastoma cells. The delivery of anti-miR-9 to the drug resistant glioblastoma cells reversed the expression of multidrug transporters and sensitized glioblastoma cells to temzolomid, as shown by increased cell death and caspase activity [97]. MiR-143 can be transferred via exosomes from MSC to osteosarcoma cells suppressing their migratory abilities [95].

In a murine sarcoma model, mice were treated with exosomes containing TGF-β1-siRNA. These exosomes strongly suppressed TGF-β1 expression and signaling in the recipient tumor cells, and thus inhibited the growth of the tumor cells and the development of lung metastases [98].

Furthermore, MSC are able to deliver conventional chemotherapeutics like Paclitaxel to tumor cells via exosomes. Pascucci and collegues demonstrated that MSC loaded with paclitaxel secrete a significant amount of paclitaxel in exosomes. The paclitaxel-containing exosomes possess strong anti-proliferative effects on human pancreatic cells [99]. Paclitaxel loaded exosomes have 50 times more cytotoxicity than free paclitaxel for drug resistant cancer cells in vitro [100, 101]. They can also reduce metastases and tumor size in a mouse model of lung carcinoma. The authors concluded that exosome-encapsulated paclitaxel might directly target drug resistant CSCs [100].

Most research on exosome-based therapy has been performed in vitro or in mouse models. However there are already a few clinical trials using exosomes. A phase I trial showed that exosomes loaded with tumor antigens were able to activate immune response and disease progression was slowed in a small number of non-small cell lung cancer patients [102]. A phase II trial was performed which showed that IFN-γ-loaded exosomes were capable of boosting NK cell-mediated anti-tumor immunity in advanced non-small cell lung cancer patients. Thirty two percent (7/22) of participants experienced stabilization for more than 4 months [103].

## Conclusion

Exosomes are functional mediators of tumor stroma interaction and play a fundamental role in every mentioned category of tumor therapy resistance.

Exosomes can mediate therapy resistance by direct drug export, intracellular reduction of drugs and by the transport of drug efflux pumps. Exosomes can shift cellular homeostasis between anti- and pro-apoptotic signalling resulting in increased tumor cell survival after exposure to DNA damaging chemotherapeutic drugs or irradiation and increase DNA repair. Moreover, exosome signaling generates therapy resistant conditions in the tumor microenvironment and induces cancer stem cell like phenotypes through EMT in tumor cells leading to environment-mediated drug resistance.

## Abbreviations

ABC transporter: ATP-binding cassette transporter
ADCC: Antibody dependent cellular cytotoxicity
CD: Cluster of differentiation
CSC: Cancer stem cell
EMT: Epithelial to mesenchymal transition
MDR genes: Multidrug resistance genes
miRNA (miR): micro RNA
mRNA: messenger RNA
MSC: Mesenchymal stem cell
NK cells: Natural killer cells
P-gp: P-glycoprotein
TGF-β1: Tumor growth factor-β1
